# Plantar Dermal Deposition of Wild-Type Transthyretin Amyloid (ATTR): A Case Report of a Unique and Challenging Histopathological Manifestation of Cutaneous ATTR Amyloidosis

**DOI:** 10.7759/cureus.84518

**Published:** 2025-05-21

**Authors:** Shojiro Ichimata, Akane Aikawa, Takayuki Ishii, Michiro Maruyama, Tsuneaki Yoshinaga, Mitsuto Sato, Nagaaki Katoh, Fuyuki Kametani, Masahide Yazaki, Yoshiki Sekijima, Shin Ishizawa

**Affiliations:** 1 Department of Legal Medicine, University of Toyama, Toyama, JPN; 2 Department of Pathology, Toyama Prefectural Central Hospital, Toyama, JPN; 3 Department of Dermatology, Toyama Prefectural Central Hospital, Toyama, JPN; 4 Department of Cardiology, Toyama Prefectural Central Hospital, Toyama, JPN; 5 Department of Medicine (Neurology and Rheumatology), Shinshu University School of Medicine, Matsumoto, JPN; 6 Department of Brain and Neurosciences, Tokyo Metropolitan Institute of Medical Science, Tokyo, JPN; 7 Institute for Biomedical Sciences, Shinshu University, Matsumoto, JPN

**Keywords:** amyloid transthyretin, collagen fiber, dermis, mechanical stress, planta

## Abstract

A 74-year-old man presented with a cutaneous lesion on the plantar surface of his right foot. His medical history included spinal canal stenosis and bilateral carpal tunnel syndrome. The lesion was clinically suspected to be a fibroma and was subsequently resected. Histopathological examination revealed mild acanthosis and hyperkeratosis, along with eosinophilic changes and thickening of collagen bundles at the base of the specimen. Based on the patient’s medical history and the pathological findings, transthyretin amyloid (ATTR) deposition was suspected. Congo red staining confirmed the presence of amyloid deposits, which were further identified as ATTR through immunohistochemistry and proteomic analysis. Genetic testing of the transthyretin gene revealed no mutations, leading to a diagnosis of wild-type ATTR (ATTRwt) amyloidosis. Cardiac function tests showed no significant abnormalities. Given that ATTRwt deposition is frequently associated with musculoskeletal disorders, amyloid may preferentially accumulate in connective tissues subjected to repetitive mechanical stress. As observed in other musculoskeletal tissues, amyloid deposition in the plantar dermis may aid in the early detection of ATTRwt. Therefore, detailed histopathological evaluation of plantar cutaneous lesions is important, especially in older adults and/or individuals with symptoms suggestive of ATTRwt amyloidosis.

## Introduction

Transthyretin amyloid (ATTR) amyloidosis is a major systemic form of amyloidosis and the most common cause of cardiac involvement, which is, in turn, the leading cause of death in systemic amyloidosis [1,2]. ATTR amyloidosis can be classified as either hereditary or wild-type (ATTRwt) [3]. Early and accurate histopathological diagnosis is essential, as tafamidis - one of the primary treatments for ATTR cardiac amyloidosis (CA) [1] - is most effective when administered in the early stages of the disease [4]. Musculoskeletal manifestations such as carpal tunnel syndrome (CTS), spinal canal stenosis (SCS), and osteoarthritis are frequently observed in patients with ATTRwt and often precede the development of cardiac symptoms [5-7]. Therefore, ATTRwt deposition in injured connective tissues, where it tends to occur, may precede cardiac involvement and serve as an early indicator of the disease. Herein, we report a case of ATTRwt deposition in the plantar dermis.

## Case presentation

**Figure 1:**
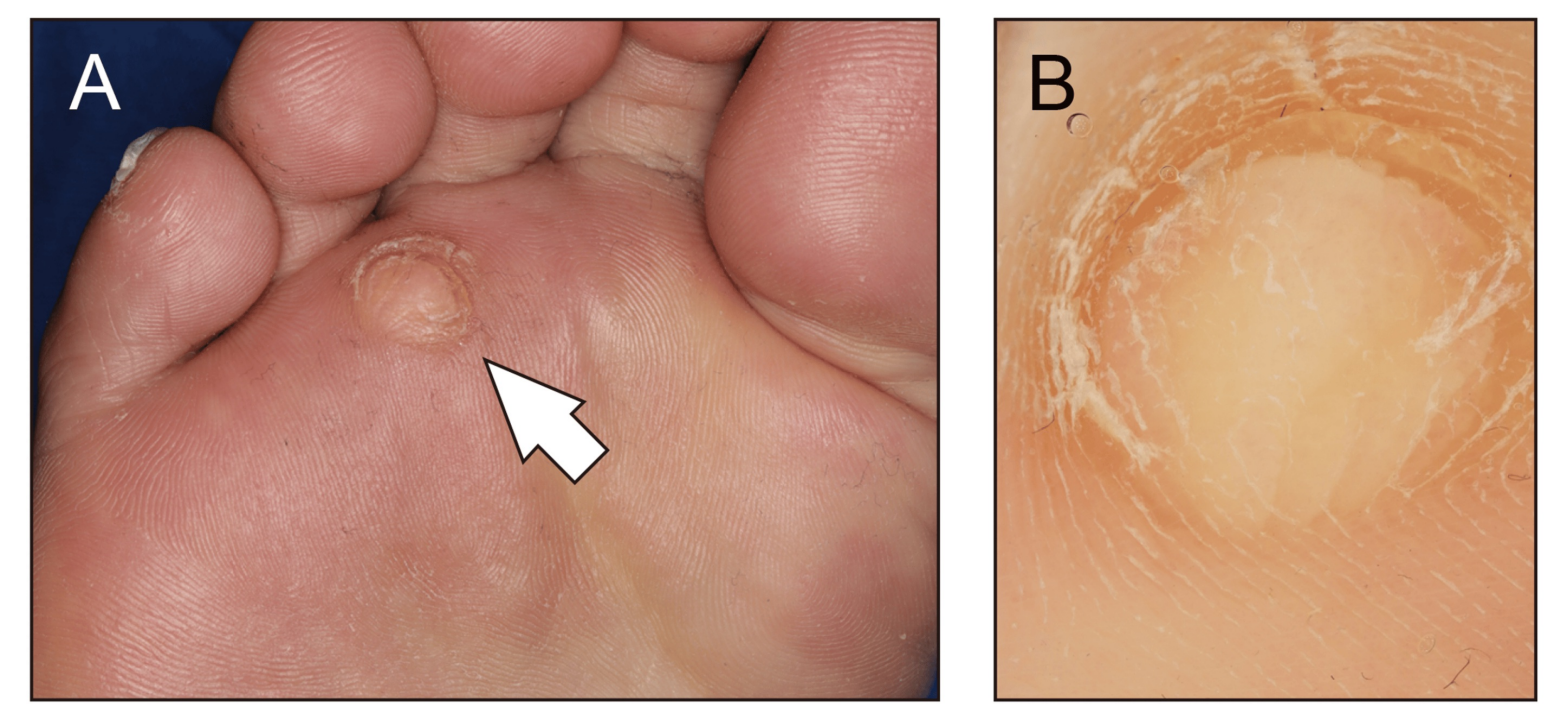
Gross (A) and dermoscopic (B) views of the cutaneous lesion The arrow in panel A indicates the lesion.

A 74-year-old man presented with a skin lesion on the plantar surface of his right foot. Although the lesion was not severely painful, it caused discomfort, and treatment with over-the-counter medications for clavi had not improved it. His medical history included surgery for SCS (approximately 20 years earlier) and bilateral CTS (details unavailable). Pathological findings from these prior surgical specimens were not available. Based on the gross and dermoscopic findings (Figure 1A, 1B), the lesion was initially suspected to be a fibroma deformed by load bearing.

**Figure 2:**
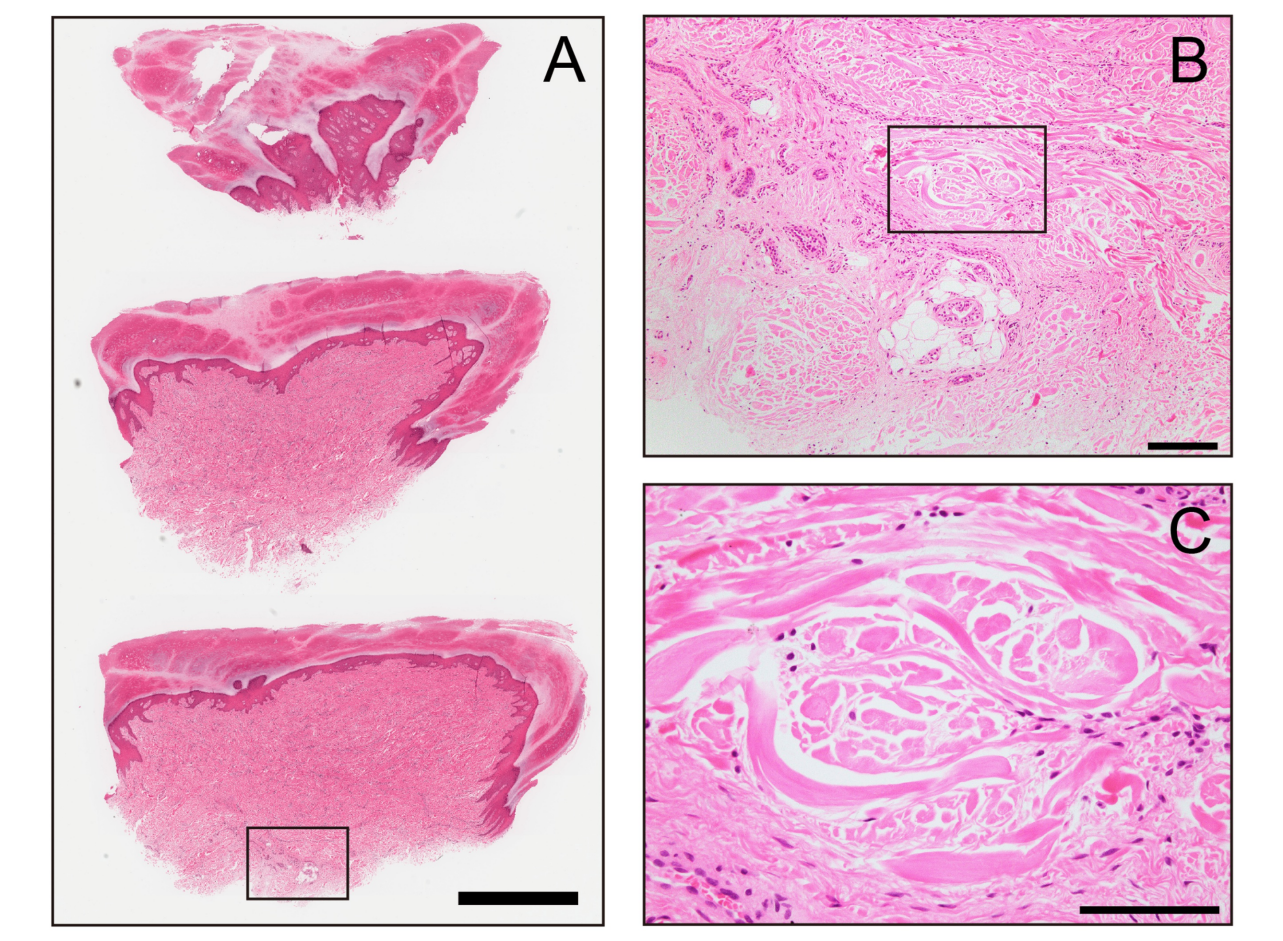
Representative histopathological findings of the cutaneous lesion in H&E-stained specimens (A-C) H&E staining. Panels B and C are magnified views of panels A and B, respectively. (A) Mild acanthosis and hyperkeratosis are observed. Additionally, collagen fibers show a mild increase within the dermis, with no distinct focal lesions suggestive of neoplastic changes. (B, C) The base of the specimen exhibits a slightly organized architecture of collagen fiber bundles, along with eosinophilic changes and thickening. Scale bars = 2 mm (A); 200 μm (B); 100 μm (C).

Subsequently, the lesion was excised. Histopathological examination of H&E-stained sections revealed hyperkeratosis and mild acanthosis (Figure 2A), with no distinct focal lesions indicative of neoplastic changes. At the base of the specimen, the collagen bundle architecture appeared slightly disorganized, showing eosinophilic changes and thickening (Figure 2B, 2C).

**Figure 3:**
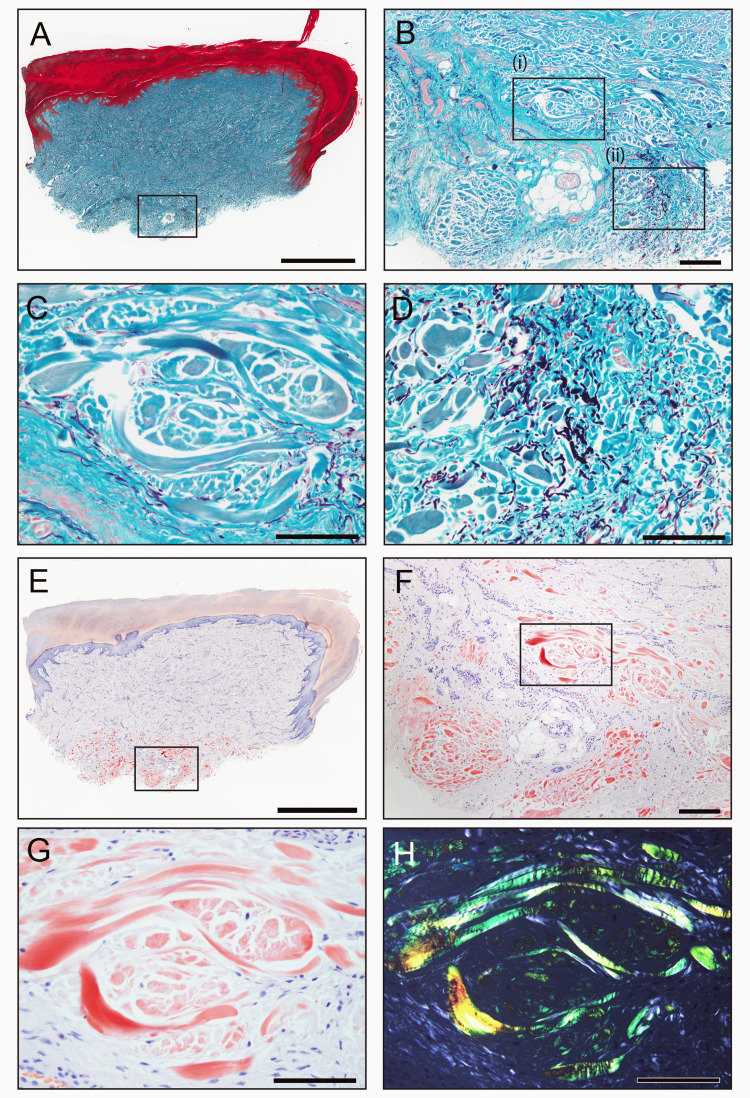
Representative histopathological findings of the cutaneous lesion using special stains (A-D) Elastica-Masson staining; (E-H) phenol Congo red staining observed under bright-field (E-G) and polarized light (H). Panels C and D are magnified views of areas (i) and (ii) in panel B, respectively. Panel G shows a higher magnification of the area in panel F. (A-D) Thickened collagen bundles at the base of the specimen appear gray-greenish. Notably, the color differs between the thickened collagen fibers on the left and the normal collagen fibers on the right (D). (E-H) These bundles are Congo red-positive, displaying apple-green birefringence under polarized light. Scale bars = 2 mm (A, E); 200 μm (B, F); 100 μm (C, D, G, H).

On Elastica-Masson staining, these bundles appeared greenish-gray, clearly distinct from the surrounding collagenous tissue (Figure 3A-3D). Based on these pathological findings and the patient’s medical history, amyloid deposition was suspected. Congo red staining confirmed amyloid deposits within these thickened bundles (Figure 3E-3H).

**Figure 4:**
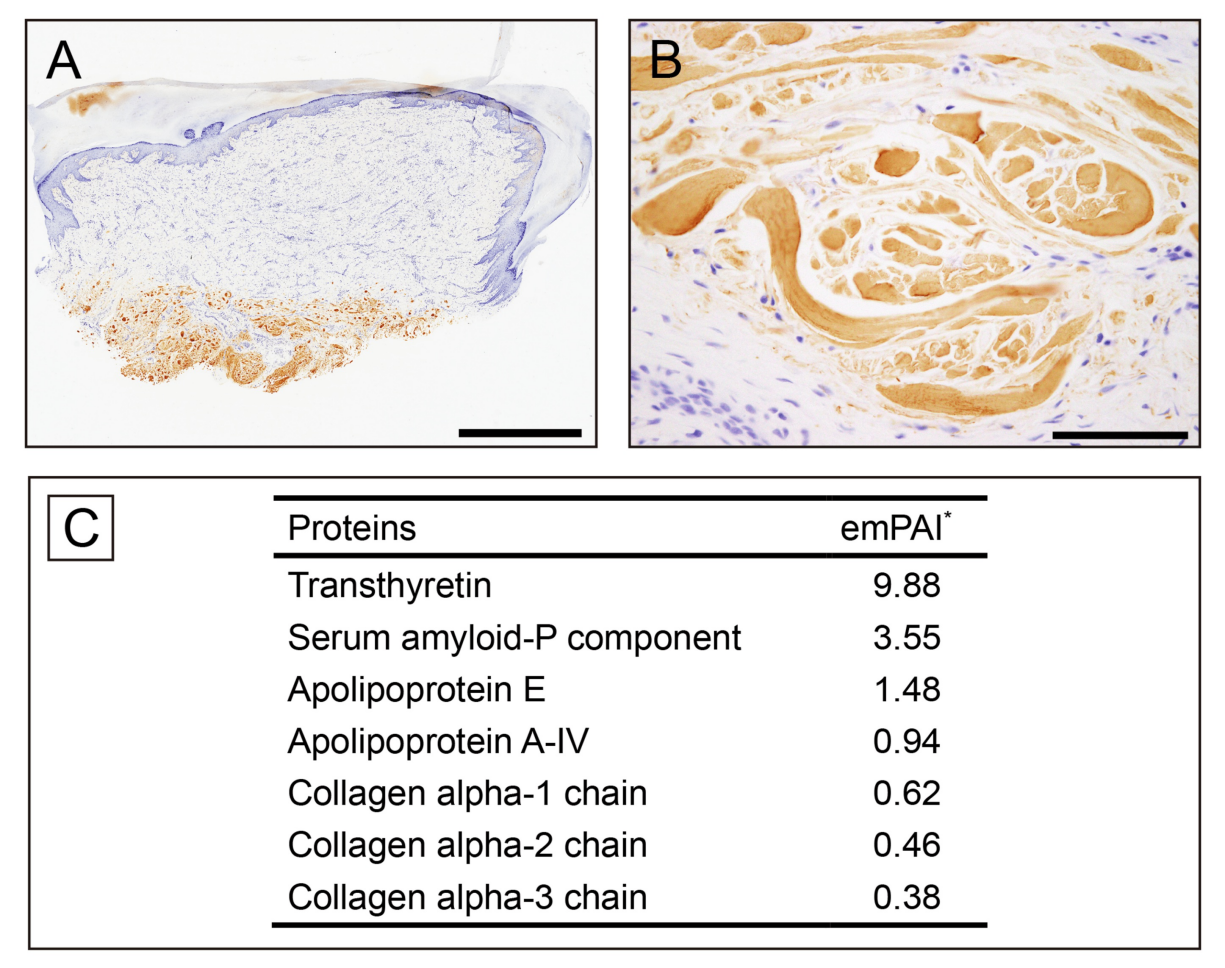
Immunohistochemical and proteomic analysis of amyloid deposition (A, B) Immunohistochemistry for prealbumin (TTR); (C) Summary of proteins identified by proteomic analysis using laser microdissection combined with liquid chromatography-tandem mass spectrometry. (A, B) Amyloid deposits show positive staining for TTR. (C) TTR is detected alongside amyloid-associated proteins. Relative protein quantification in the mass spectrometry-based proteomic analysis was estimated using the exponentially modified protein abundance index. Scale bars = 2 mm (A); 100 μm (B). TTR, transthyretin

No amyloid deposition was identified in the vessel walls. Immunohistochemically, the amyloid deposits tested positive for transthyretin (TTR) (Figure 4A, 4B) and were negative for immunoglobulin κ and λ light chains as well as amyloid A.

Table 1 summarizes the immunohistochemical methods used in this study.

**Table 1:** Summary of antibodies and immunohistochemistry methods used in this study Immunostaining was performed using the Leica Bond-IV automated system and Leica Refine detection kits (Leica Biosystems, Bannockburn, IL, USA) following the manufacturer’s instructions. All sections were subsequently counterstained with hematoxylin.

Antibody	Source	Clone	Dilution	Antigen retrieval
Igλ	Abcam (Cambridge, UK)	Polyclonal	0.736111	100% formic acid (one minute)
Igκ	DB Biotech (Košice, Slovakia)	H16-E	0.388889	100% formic acid (one minute)
Amyloid A	Dako (Glostrup, Denmark)	MC1	2.819444	None
Prealbumin	Abcam (Cambridge, UK)	EPR3219	1.430556	100% formic acid (one minute)

The amyloidosis consultation system in Japan further confirmed these findings [8]. For additional characterization, proteomic analysis using laser microdissection and liquid chromatography-tandem mass spectrometry was performed as previously described [9]. The results confirmed TTR deposition along with coexisting amyloid-associated proteins (Figure 4C).

Notably, apolipoprotein A-I, which is frequently detected in amyloid deposits in other musculoskeletal tissues [10], was not identified. However, components of collagen fibers were detected alongside the amyloid deposits, confirming that these deposits formed on the collagen fibers. Following the pathological diagnosis, TTR gene analysis was performed and revealed no mutations. Thus, the patient was diagnosed with ATTRwt amyloidosis. Cardiac function tests were also conducted and showed no abnormalities. The patient has been followed as an outpatient, with no apparent cardiac symptoms at one year postoperatively.

## Discussion

This case report highlights a distinctive histopathological pattern of ATTRwt deposition along collagen bundles in the plantar dermis. The clinical course and histopathological findings suggest that the lesion was subjected to prolonged mechanical stimulation. Chronic mechanical stress is a key factor in the development of musculoskeletal disorders associated with ATTRwt amyloidosis [11]. Marcoux et al. demonstrated that mechanical stress, particularly shear stress, plays a crucial role in TTR amyloidogenesis [12]. Moreover, some amyloid deposits, including ATTR, have been linked to type I and type II collagens [13], both of which were detected alongside amyloid in this study. Taken together, connective tissue degeneration - especially involving type I and type II collagens - along with TTR tetramer cleavage and dissociation triggered by chronic mechanical stimulation, may underlie ATTR deposition. Additionally, Misumi et al. reported that fibroblasts can endocytose denatured TTR aggregates [14], which might also relate to the distinctive deposition pattern observed here.

However, in this case, it remains unclear whether ATTR deposition on collagen fibers denatured by repetitive stimulation led to the formation of the protruded lesion or whether ATTR deposition itself induced collagen fiber degeneration and hyperplasia, resulting in lesion formation. To the best of our knowledge, reports of ATTRwt deposition forming protruded cutaneous lesions, as seen in this case, are lacking. Thus, the latter hypothesis appears less likely, but further investigation is warranted. Moreover, these patients may be prone to recurrent plantar lesions; therefore, preventing continuous weight bearing on specific plantar areas, similar to strategies used for corn prevention, is important.

It is well-known that ATTRwt deposits are frequently found in surgical specimens from patients with musculoskeletal disorders such as CTS and SCS [5-7,10,13]. Notably, this patient had a history of both SCS and CTS. Although pathological data from those surgical sites were unavailable, ATTR deposits may have been present there. This case suggests that some patients may be predisposed to ATTR deposition in degenerated connective tissue following physical stimuli. Furthermore, Milandri et al. reported that CTS is an incremental risk factor for ATTR CA, especially in both ATTRwt and hereditary ATTR with cardiogenic mutations, often preceding CA diagnosis by five to nine years [7]. Therefore, patients with dermal ATTR deposits may also be at risk of developing ATTR-CA later, underscoring the need for long-term follow-up.

As shown in this study, ATTR deposits localize along collagen fiber bundles, which can make histopathological identification on H&E-stained specimens challenging. In addition, no ATTR deposition was observed in vessel walls, a typical site of ATTR accumulation [15]. These factors complicate diagnosis and may explain why cutaneous lesions related to ATTRwt amyloidosis remain underreported. In this case, ATTR deposition might have gone unrecognized without knowledge of the patient’s CTS and SCS history. Thus, surgical specimens from the plantar region in older adults (≥60 years) with symptoms suggestive of ATTR amyloidosis, such as cardiac manifestations or specific musculoskeletal signs, should be carefully examined histopathologically. Congo red staining should be performed alongside H&E staining to avoid missing ATTR deposits. However, it is important to note that congophilia in ATTRwt deposits is generally weak [16], and Congo red staining may yield nonspecific positivity in collagen fibers. In such cases, special stains like Masson’s trichrome or Elastica van Gieson may help differentiate amyloid from connective tissue, while immunohistochemistry for TTR can provide a definitive diagnosis.

## Conclusions

This report describes a distinctive pattern of ATTRwt deposition in the plantar dermis. Connective tissues in the plantar region, which are subjected to continuous mechanical stimulation, may be particularly susceptible to ATTR deposition. This suggests that ATTR deposits could potentially complicate common plantar disorders caused by mechanical stress, such as calluses or clavus. Therefore, such lesions might serve as useful biopsy targets for detecting ATTRwt deposition. Future studies should explore this possibility, especially since histopathological examination is rarely performed on specimens from these lesions.

Similar to certain musculoskeletal conditions, ATTRwt deposition along dermal collagen fibers may precede the onset of cardiac symptoms, making it potentially valuable for identifying early-stage CA. Given the limited knowledge about the symptoms, treatment, and prevention of cutaneous ATTRwt deposition, further research is necessary. Importantly, diagnosis based solely on H&E staining is challenging. Multiple staining techniques, including special stains and TTR immunohistochemistry, are essential for accurate detection. Additionally, detailed clinical information, such as the presence of cardiac symptoms or musculoskeletal diseases, even without a surgical history, is critical to raise histopathological suspicion of ATTR deposition. Therefore, close collaboration and information sharing between pathologists and clinicians are vital to improving the detection and management of cutaneous ATTR deposition.

## References

[1] Wechalekar AD, Gillmore JD, Hawkins PN (2016). Systemic amyloidosis. Lancet.

[2] Ruberg FL, Maurer MS (2024). Cardiac amyloidosis due to transthyretin protein: a review. JAMA.

[3] Buxbaum JN, Eisenberg DS, Fändrich M (2024). Amyloid nomenclature 2024: update, novel proteins, and recommendations by the International Society of Amyloidosis (ISA) Nomenclature Committee. Amyloid.

[4] Maurer MS, Schwartz JH, Gundapaneni B (2018). Tafamidis treatment for patients with transthyretin amyloid cardiomyopathy. N Engl J Med.

[5] Aldinc E, Campbell C, Gustafsson F (2023). Musculoskeletal manifestations associated with transthyretin-mediated (ATTR) amyloidosis: a systematic review. BMC Musculoskelet Disord.

[6] Westin O, Fosbøl EL, Maurer MS (2022). Screening for cardiac amyloidosis 5 to 15 years after surgery for bilateral carpal tunnel syndrome. J Am Coll Cardiol.

[7] Milandri A, Farioli A, Gagliardi C (2020). Carpal tunnel syndrome in cardiac amyloidosis: implications for early diagnosis and prognostic role across the spectrum of aetiologies. Eur J Heart Fail.

[8] Abe R, Katoh N, Takahashi Y (2021). Distribution of amyloidosis subtypes based on tissue biopsy site - consecutive analysis of 729 patients at a single amyloidosis center in Japan. Pathol Int.

[9] Kametani F, Haga S (2015). Accumulation of carboxy-terminal fragments of APP increases phosphodiesterase 8B. Neurobiol Aging.

[10] Tasaki M, Lavatelli F, Obici L (2021). Age-related amyloidosis outside the brain: a state-of-the-art review. Ageing Res Rev.

[11] Ballestero-Pérez R, Plaza-Manzano G, Urraca-Gesto A (2017). Effectiveness of nerve gliding exercises on carpal tunnel syndrome: a systematic review. J Manipulative Physiol Ther.

[12] Marcoux J, Mangione PP, Porcari R (2015). A novel mechano-enzymatic cleavage mechanism underlies transthyretin amyloidogenesis. EMBO Mol Med.

[13] Yanagisawa A, Ueda M, Sueyoshi T (2016). Knee osteoarthritis associated with different kinds of amyloid deposits and the impact of aging on type of amyloid. Amyloid.

[14] Misumi Y, Ando Y, Gonçalves NP, Saraiva MJ (2013). Fibroblasts endocytose and degrade transthyretin aggregates in transthyretin-related amyloidosis. Lab Invest.

[15] Pinton S, Vacchi E, Chiaro G (2023). Amyloid detection and typing yield of skin biopsy in systemic amyloidosis and polyneuropathy. Ann Clin Transl Neurol.

[16] Bergström J, Gustavsson A, Hellman U (2005). Amyloid deposits in transthyretin-derived amyloidosis: cleaved transthyretin is associated with distinct amyloid morphology. J Pathol.

